# Exploring Better Strategies for RAS Mutation-Associated EGFR-Targeted Resistance in Colorectal Cancer: From the Perspective of Cancer Community Ecology

**DOI:** 10.3389/fonc.2021.754220

**Published:** 2021-10-22

**Authors:** Xiaojie Wang, Wenchuan Wu, Zhifang Zheng, Pan Chi

**Affiliations:** ^1^ Department of Colorectal Surgery, Union Hospital, Fujian Medical University, Fuzhou, China; ^2^ Department of General Surgery, Zhongshan Hospital, Fudan University, Shanghai, China

**Keywords:** EGFR, resistance, RAS, cancer community ecology, colorectal cancer

## Abstract

RAS is the most common mutated gene in colorectal cancer (CRC), and its occurrence is associated with primary and acquired resistance to anti-epidermal growth factor receptor (EGFR) blockade. Cancer community ecology, such as the competitive exclusion principle, is a valuable focus and would contribute to the understanding of drug resistance. We have presented several articles on RAS mutant clonal evolution monitoring during anti-EGFR treatment in CRC. In these articles, the availability of serially collected samples provided a unique opportunity to model the tumor evolutionary process from the perspective of cancer community ecology in those patients upon treatment. In this perspective article, we presented a theoretical basis and evidence from several experimental or phase II clinical trials for the contemporary application of ecological mechanisms in CRC treatment. In general, a reduction in targetable RAS wild-type cells to a maximum tolerated extent, such as continuous treatment, might lead to the competitive release of inextirpable RAS mutant cells and cancer progression. A full understanding of subclonal competition might be beneficial in managing CRC. Several ecological strategies, including anti-EGFR treatment reintroduced at an appropriate point of time for RAS mutant patients, intermittent treatment instead of continuous treatment, the appropriate sequence of nonselective targeted therapy, and combination therapy, were proposed.

## Introduction

As a critical element of the mitogen activated protein kinase (MAPK) pathway, RAS is the most common mutated gene in colorectal cancer (CRC), and its occurrence is associated with a lack of response to anti-epidermal growth factor receptor (EGFR) blockade. Moreover, a large fraction of patients with KRAS wild-type metastatic CRC achieve an initial response to cetuximab or panitumumab and then progress after 3-12 months. The molecular alterations (in most instances, mutations of RAS) are causally responsible for acquired resistance to anti-EGFR treatment (1). We have read with great interest several articles on RAS mutant clonal evolution monitoring during anti-EGFR treatment. In these articles, the availability of serially collected samples provided a unique opportunity to model the tumor evolutionary process from the perspective of cancer community ecology in those patients upon treatment.

## Competitors: Subclonal Competition Between Mutant RAS Cells and RAS Wild-Type Cells

We are familiar with the mutation heterogeneity across metastatic deposits or primary tumors. A previous study indicated that 11.3% of patients with mutant KRAS primary tumors had wild-type KRAS in the metastases (2). This represented the frequency of the loss of opportunity for receiving potentially beneficial anti-EGFR treatment. In turn, less is understood about the genetic heterogeneity in subclones within the primary tumor. Since patients whose CRCs were initially RAS wild-type developed detectable RAS mutations in their sera during EGFR therapies, it is still unclear whether the acquired resistance is due to the selection of pre-existing resistant clones under drug pressure or truly therapy-induced resistant clones. A mathematically proven hypothesis to explain the development of resistance to EGFR therapies is that rare cells (one in ~42) with RAS mutations pre-exist at low levels in tumors with ostensible wild-type RAS genes (3). Conversely, not all cells carried RAS mutations in the ostensibly RAS mutation population. Direct evidence supporting the pre-existence of mutant RAS clones in RAS wild-type tumors comes from an early clinical histological study. Remarkable intratumor heterogeneity before chemotherapy was confirmed, where different KRAS mutation statuses between the tumor center region and the margin were detected with a high percentage of 44% (4). Other indirect evidence is that the genetic landscape of secondary resistance to EGFR therapies partially overlaps with that of primary resistance (5). According to the competitive exclusion principle, when different cancer cell species, such as mutant RAS cells and RAS wild-type cells, coexist within the same tumor microenvironment, they form an ecological community and compete for the same set of resources (6). Although the clearance of RAS mutations is a rare event (7), it is conceivable that subclones with RAS mutations are less fit in the untreated tumor and acquire fitness as a consequence of adaptation to the microenvironment induced by EGFR therapies.

**Figure 1:**
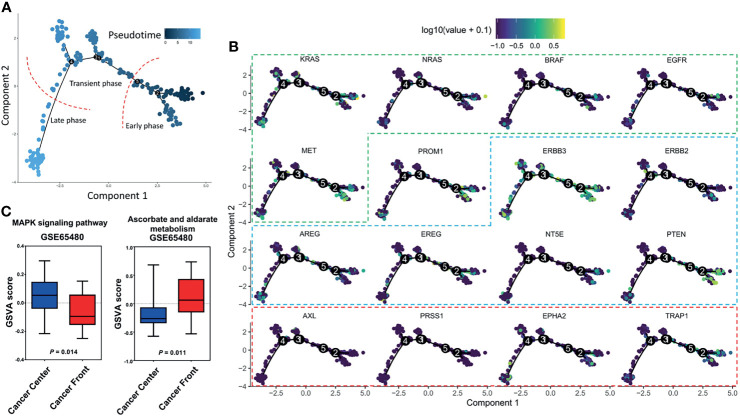
Trajectory analysis of single-cell transcriptomes within colorectal cancers from GSE81861. **(A)** Cells following the development trajectory were classified as early, transient and late phases. **(B)** RNA expression of genes across the pseudotime trajectory. Genes from the EGFR signaling axis (KRAS, NRAS, BRAF, EGFR, and MET) are shown in the green frame. EGFR-sensitive genes (ERBB3, ERBB2, EREG, AREG, NT5E, and PTEN) are shown in the blue frame. EGFR-resistance genes (TRAP1, AXL, PRSS1, and EPHA2) are shown in the red frame. **(C)** Gene set variation analysis of bulk samples between the tumor front and the center.

To better understand the landscape of intratumor heterogeneity, we utilized a collection of single-cell transcriptomes within CRC tumors from GSE81861 (8) and performed a trajectory analysis to order 561 CRC cells in developmental pseudotime using R version 3.6.2. R package Seurat package was used to process the single-cell sequencing data, then R package monocle was used to conduct pseudotime analysis. The cells were reduced dimensionality by the DDRTree method, sequenced and visualized in pseudotime. Cells following the development trajectory were classified as early, transient and late phases (**Figure 1A**). Then, the RNA expression of genes from the EGFR signaling axis (KRAS, NRAS, BRAF, EGFR, and MET), a tumor stem cell marker (PROM1), EGFR-resistance genes (TRAP1, AXL, PRSS1, and EPHA2) and EGFR-sensitive genes (ERBB3, ERBB2, EREG, AREG, NT5E, and PTEN, summarized in **Table 1**) were mapped across the pseudotime trajectory (**Figure 1B**). The intratumor expression pattern is heterogeneous across the pseudotime trajectory. For genes from the EGFR signaling axis, KRAS was widely distributed on the pseudotime trajectory, while NRAS, BRAF, and EGFR were mainly enriched in the early phase of the trajectory and were only expressed in a small subset of subclones. The cells with high PROM1 (CD133) expression were concentrated at the early phase of the cell trajectory, with a small subset in the late phase. Interestingly, the cell clones with high expression of EGFR-resistant genes accounted for a very small proportion of untreated CRC samples and were unevenly distributed across the pseudotime trajectory. The expression levels of AXL and PRSS1 were persistently low. EPHA2 expression was widely distributed, although at a low level, in the whole pseudotime, while TRAP1 was mainly concentrated at the early phase. Similar expression patterns were observed in EGFR-sensitive genes. The above results provide evidence of the molecular heterogeneity of resistant/sensitive clones in CRC along the pseudotime trajectory. Thus, narrow-spectrum targeted therapy will never eradicate all resistant clones.

**Table 1:** Anti-EGFR sensitive/resistant genes at expression level.

Gene	Drugs Affected	anti-EGFR sensitive/resistant	Expression-Consequences	Trial	References
ERBB3	Panitumumab	Sensitive	Upregulation-higher PFS	PICCOLO	(9)
ERBB2	Cetuximab	Sensitive	Upregulation-higher survival	CALGB 80202	(10)
AREG	Panitumumab	Sensitive	Upregulation-higher PFS	PICCOLO	(11)
EREG	Panitumumab	Sensitive	Upregulation-higher PFS	PICCOLO	(11)
NT5E	Cetuximab	Sensitive	Upregulation-higher survival	CALGB 80203	(10)
PTEN	Cetuximab	Sensitive	Loss of expression-nonresponsiveness		(12)
AXL	Cetuximab	Resistant	Upregulation-poor PFS, OS		(13)
PRSS1	Cetuximab	Resistant	Upregulation-poor PFS		(14)
EPHA2	Cetuximab	Resistant	Upregulation-poor PFS; increased progression rate	CAPRI-GOIM	(15)
TRAP1	Cetuximab	Resistant	Upregulation-poor response		(16)

PFS, progression-free survival; OS, overall survival.

Clinical evidence of subclonal competition comes from an extreme clinical condition. A multicenter phase 2 single-arm trial assessed the activity of the rechallenge strategy with cetuximab as third-line treatment for patients with RAS wild-type metastatic CRC (n=28) who were initially sensitive to and then resistant to first-line cetuximab-based therapy. The results showed an overall response rate of 21% and a disease control rate of 54% (17). During the first-line treatment, cetuximab selectively reduces the sensitive (wild-type) clones, thus making the resistant (mutant) cells gradually predominant until tumor progression. During the second-line non-cetuximab-based treatment, RAS wild-type clones would be partially restored, thus making them reactive to anti-EGFR rechallenge. A similar phenomenon was observed in another group of 7 patients with initial RAS-mutant metastatic CRC converted to RAS wild-type status in plasma at the time of progressive disease from bevacizumab-containing treatments. All patients benefited from subsequent anti-EGFR treatment (18). Although the resistant clones were difficult to completely eradicate, the subclonal competition theory between sensitive and resistant clones may have implications in tumor control at the macro level, which provides opportunities for receiving potentially beneficial anti-EGFR treatment for a subset of initial or acquired RAS-mutant metastatic CRCs.

## Food-Safety Tradeoffs: Implications for Designing Treatment Frequency and Sequence

Communities in nature constantly see the coexistence of a species that is a more effective competitor for resources but that is less defensive to predators and one that is better able to avoid predators at the cost of being efficient in obtaining resources (Food-Safety Tradeoffs) (6). Subclones with RAS mutations are impervious to anti-EGFR-targeted therapies and chemotherapeutic attack at a metabolic cost in regions with standard glucose conditions (19), which restricts their clonal expansion as a result of inhibition by RAS wild-type cells in an untreated tumor. Such a mechanism of coexistence may be beneficial in strategically designing the frequency and sequence of treatment.

In an interesting exploratory randomized phase 2 trial (COIN-B) (20), patients with KRAS wild-type advanced CRC were assigned to the intermittent cetuximab group (n=78) or continuous cetuximab group (n=91). Patients in both groups first received 12 weeks of FOLFOX and concurrent weekly cetuximab. Then, in the intermittent cetuximab group, chemotherapy and cetuximab were stopped until tumor progression. In the maintenance cetuximab group, patients continued with weekly cetuximab, and only on tumor progression was FOLFOX reintroduced. The primary outcome was failure-free survival at 10 months, which was met for both groups (50% for the intermittent cetuximab group and 52% for the continuous cetuximab group). From the perspective of cancer community ecology, this is in support of the hypothesis that intermittent treatment might inhibit RAS mutant clones through sensitive RAS wild-type clones and control tumor burden at least as effective as continuous treatment.

Another randomized phase II study (REVERCE) challenged the standard therapeutic sequence of cetuximab followed by regorafenib for metastatic CRC (21). Patients with KRAS exon 2 wild-type metastatic CRC after the failure of fluoropyrimidine, oxaliplatin, and irinotecan were randomized to receive sequential treatment with regorafenib followed by cetuximab (R-C arm, n=51) or the reverse sequence (C-R arm, n=50). The study was designed as a non-inferiority trial. For the primary endpoint, the median overall survival (OS) in the R-C arm was longer than that in the C-R arm (17.4 *vs.* 11.6 months, P = 0.0293). Key secondary endpoints included progression-free survival (PFS) with initial treatment (PFS1) and PFS with second treatment (PFS2). Interestingly, no significant difference was observed in PFS1 between the two arms, whereas PFS2 was superior in the R-C arm (median PFS2, R-C arm *vs.* C-R arm: 5.2 *vs.* 1.8 months, P<0.0001). After the failure of first-line therapy, emerging RAS mutations were observed in only 1 patient after regorafenib (R-C arm) compared to 11 patients after cetuximab (C-R arm). This study provides proof-of-principle that continuous first-line anti-EGFR treatment “selects” for RAS mutant clones to survive and results in resistance to further second-line treatment. This is reflected in the higher frequency of RAS mutations observed after cetuximab treatment and the worse PFS2 of the C-R arm than the R-C arm.

## Diet Choice: Implications for Combination Therapy

The tradeoff necessary for coexistence is that to be more competitive in obtaining one type of food, a species sacrifices efficiency with another type of food (diet choice) (6). Interestingly, this diet choice is affected by the abundance of resources in the ecological environment. Thus, each species with a different diet choice adapts to its specific habitat, which contributes to habitat heterogeneities (habitat selection). RAS wild-type cells require EGF as an essential resource, whereas RAS mutant cells are independent of EGF. In low-glucose conditions, the RAS mutant cells increased glucose transporter 1 (GLUT1) expression to guarantee their survival, whereas very few cells with wild-type KRAS alleles survived when they were subjected to a low-glucose environment (19). Again, the increase in the ability to grab resources comes at the expense of drug resistance. For instance, RAS mutant cells were more vulnerable to oxidative stress than RAS wild-type cells, as RAS mutant cells were selectively killed when exposed to high levels of vitamin C (ascorbate) due to increased uptake of the oxidized form of vitamin C *via* the GLUT1 glucose transporter. It is known that the mechanisms of secondary resistance to anti-EGFR biochemically converge to constitutive activation downstream of the EGFR-RAS-MAPK pathway (5). To compare the activity of pathways between the tumor front and the center, we performed a gene set variation analysis (GSVA) based on 20 pairs of untreated CRC clinical bulk samples from GSE65480 (22) to compute the pathway enrichment scores using the R package GSVA with default parameters. Interestingly, the results showed that different MAPK signaling pathway activities and ascorbate and aldarate metabolism levels were identified between the tumor front and the center (**Figure 1C**), which supports the habitat selection theory in the untreated tumor. The above phenomena provide a mechanistic rationale for exploring the combined use of anti-EGFR and vitamin C therapies to directly target both the essential resources themselves (EGF) and the pivotal player involved in obtaining resources (glucose transporter) for CRC. Furthermore, this hypothesis has recently been tested in CRC patient-derived xenografts. Cetuximab in combination with vitamin C could restrain and delay the emergence of secondary resistance to EGFR blockade in CRC RAS/BRAF wild-type models (23).

A limitation of this study is that we only employed RAS mutational status as an example of how cancer community ecology theory can explain the anti-EGFR resistance. Other potential explanations for the mechanism of anti-EGFR resistance, such as human epithelial growth factor receptor-2 (HER2) amplification (24), were not discussed. In addition, a total of 3 phase II studies were presented to provide proof-of-principle evidence on the value of ecological strategies. It is worth noting that all these 3 studies only had small sample sizes and with single-stage design as a potential target of interest for future studies (see **Supplement Table 1** for study designs).

We provide a theoretical basis and evidence from several experimental or phase II clinical trials for the contemporary application of ecological mechanisms in CRC treatment. In general, a total of 3 cancer community ecological mechanisms, including competitors or subclonal competition theory, food-safety tradeoffs and diet choice theory, were proposed and discussed. A reduction in targetable RAS wild-type cells to a maximum tolerated extent, such as continuous treatment, might lead to the competitive release of inextirpable RAS mutant cells and cancer progression. A full understanding of subclonal competition might be beneficial in managing CRC. Several ecological strategies, including anti-EGFR treatment reintroduced at an appropriate point of time for RAS mutant patients, intermittent treatment instead of continuous treatment, the appropriate sequence of nonselective targeted therapy, and combination therapy, warrant further confirmation.

## Data Availability Statement

The original contributions presented in the study are included in the article/**Supplementary Material**. Further inquiries can be directed to the corresponding authors.

## Author Contributions

XW and WW conceived and designed the study. XW, WW, ZZ, and PC contributed to the computational analyses and confirmed the results. All authors contributed to the article and approved the submitted version.

## Funding

This study was financially supported by the National Natural Science Foundation of China (81902378).

## Conflict of Interest

The authors declare that the research was conducted in the absence of any commercial or financial relationships that could be construed as a potential conflict of interest.

## Publisher’s Note

All claims expressed in this article are solely those of the authors and do not necessarily represent those of their affiliated organizations, or those of the publisher, the editors and the reviewers. Any product that may be evaluated in this article, or claim that may be made by its manufacturer, is not guaranteed or endorsed by the publisher.
