# Supplementary material for: Exploring Better Strategies for RAS Mutation-Associated EGFR-Targeted Resistance in Colorectal Cancer: From the Perspective of Cancer Community Ecology
Source: Front Oncol. 2021 Oct 22;11:754220. doi: 10.3389/fonc.2021.754220 (PMC8568953; doi:10.3389/fonc.2021.754220)
Supplement: Supplementary file 1 [file Table_1.doc]

Supplement table 1 Study design of 3 phase II studies presented in this perspective article

| Study | Number of patients | Primary end point | Basis of the sample size and sample size calculation | Posthoc power analysis | Statistical test | Journal |
| --- | --- | --- | --- | --- | --- | --- |
| Cremolini et al [1] (data from The CRICKET trial) | Single-arm (n=28) | Overall response rate | According to the Fleming single-stage design, selecting P = .05 (overall response rate in the null hypothesis) based on results with second-line irinotecan-based therapies and selecting P = .20 (overall response rate in the alternative hypothesis) as a potential target of interest for future studies, with α (1-sided) errors of .05 and β errors of .20. A total of 27 patients were required. | Yes | NR | JAMA oncology |
| COIN-B [2] | The intermittent Cetuximab group (n=78) and the continuous cetuximab group (n=91) | Failure-free survival at 10 months | In the MRC FOCUS trial, failure-free survival at 9 months was roughly 50% for patients treated with continuous oxaliplatin and infused fluorouracil chemotherapy. COIN-B was designed as two separately powered phase 2 trials, using A'Hern's single-stage design to distinguish between a 10-month failure-free survival of 50% (implying that the treatment would be worth pursuing in a phase 3 trial, feasibility and toxic effects permitting) and of 35% (implying that the treatment would not be worth pursuing). The trial was not powered for comparison between the treatment groups. The original design aimed to recruit 136 patients (irrespective of KRAS status, 68 per group) with a one-sided α of 5% and 80% power. During the trial's suspension, interim data from COIN and COIN-B suggested that attrition before 12 weeks was 16% because of toxic effects or absence of benefit. The target enrolment was changed so that 158 patients with KRAS wild-type would be included, of whom 136 were expected to be assessable for the primary outcome. | No | Kaplan-Meier method to assess failure-free survival | The Lancet Oncology |
| REVERCE [3] | Regorafenib followed by cetuximab (R-C arm, n=51) and the reverse sequence (C-R arm, n=50) | Overall survival | This study was designed to estimate a hazard ratio (HR) between the two arms; a follow-up non-inferiority study would be planned if an observed HR was between 0.80 and 1.25, while a superiority, phase III study would be planned if an observed HR was <0.8. Ensuring 80% probability that an observed HR was between 0.80 and 1.25, under the true HR of 1.0, required 132 deaths and 180 patients in both arms. The enrolment for this study was discontinued because of slower than expected accrual and short of funding. | No | Survival curves were estimated by the Kaplan–Meier method, and the Cox proportional hazards model was used to estimate the HR. | Annals of oncology |

Reference

[1] Cremolini C, Rossini D, Dell'Aquila E, Lonardi S, Conca E, Del Re M, Busico A, Pietrantonio F, Danesi R, Aprile G *et al*: **Rechallenge for Patients With RAS and BRAF Wild-Type Metastatic Colorectal Cancer With Acquired Resistance to First-line Cetuximab and Irinotecan: A Phase 2 Single-Arm Clinical Trial**. *JAMA oncology* 2019, **5**(3):343-350.

[2] Wasan Harpreet, Meade Angela M., Adams Richard, Wilson Richard, Pugh Cheryl, Fisher David, Sydes Benjamin, Madi Ayman, Sizer Bruce, Lowdell Charles *et al*: **Intermittent chemotherapy plus either intermittent or continuous cetuximab for first-line treatment of patients with KRAS wild-type advanced colorectal cancer (COIN-B): a randomised phase 2 trial**. *The Lancet Oncology* 2014, **15**(6):631-639.

[3] Shitara K, Yamanaka T, Denda T, Tsuji Y, Shinozaki K, Komatsu Y, Kobayashi Y, Furuse J, Okuda H, Asayama M *et al*: **REVERCE: a randomized phase II study of regorafenib followed by cetuximab versus the reverse sequence for previously treated metastatic colorectal cancer patients**. *Annals of oncology : official journal of the European Society for Medical Oncology* 2019, **30**(2):259-265.
